# Association of COL5A1 gene polymorphisms and musculoskeletal soft tissue injuries: a meta-analysis based on 21 observational studies

**DOI:** 10.1186/s13018-022-03020-9

**Published:** 2022-03-03

**Authors:** Rui Guo, Zhe Ji, Shutao Gao, Aihaiti Aizezi, Yong Fan, Zhigang Wang, Kai Ning

**Affiliations:** 1 Department of Orthopedic Center, People’s Hospital of Xinjiang Uygur Autonomous Region, No.91 Tianchi Road, Urumqi, 830001 Xinjiang China; 2 Department of Spine Surgery, Xinjiang Medical University First Affiliated Hospital, Urumqi, 830054 Xinjiang China

**Keywords:** COL5A1, Polymorphism, Musculoskeletal soft tissue injury, Meta-analysis

## Abstract

**Objective:**

Inconsistent findings existed on the correlation of collagen type V α1 (COL5A1) gene polymorphisms and musculoskeletal soft tissue injuries (MSTIs). The purpose of this study was to collect and combine the current evidences by a meta-analysis approach.

**Methods:**

Six online databases were searched up to August, 2021. The methodological quality of each individual study was evaluated based upon Newcastle–Ottawa Scale (NOS). The strength of the effect size was presented by odds ratio (OR) with 95% confidence interval (95%CI) in five genetic models. The data were analyzed using Review Manager 5.3.

**Results:**

Twenty-one studies were eligible to this meta-analysis. The study quality was deemed fair to excellent according to NOS. In the overall analyses, the merged data suggested that rs12722, rs71746744, and rs3196378 polymorphisms were correlated to an increased susceptibility to MSTIs. But the association was not established in rs13946 or rs11103544 polymorphism. For rs12722 polymorphism, stratified analyses by injury type and ethnicity identified the association mainly existed in ligament injury and among Caucasian population. For rs13946 polymorphism, subgroup analysis suggested the association existed in tendon and ligament injuries.

**Conclusion:**

This study supports that rs12722 is associated with an elevated susceptibility to ligament injury, especially in the Caucasian population. Rs13946 polymorphism appears to increase the risk to tendon and ligament injuries. Rs71746744 and rs3196378 polymorphisms have a tendency to confer an elevated risk to MSTIs. However, no relevance is found between rs11103544 polymorphism and MSTIs.

## Introduction

Musculoskeletal soft tissues, including tendon, ligament, and muscle, are commonly injured as a result of participation in competitive and recreational activities. It has been estimated that over 100 million musculoskeletal soft tissue injuries (MSTIs) occur annually around the world [1]. Take Achilles tendinopathy as an example, the lifetime incidence of this disorder is nearly 10% in the general population and 50% among the elite athletes [2, 3]. MSTIs have a negative impact on the quality of life. Affected individuals always suffer from discomfort, pain or incapacity. For the athletes, MSTIs may lead to significant loss of sporting performance and a premature end to their careers. The management of MSTIs is difficult, thus imposing a substantial burden on society.

A fully recognition on the etiology of MSTIs is of great important to prevent these injuries. Nevertheless, the etiology of MSTIs is multifactorial and its pathogenesis remains largely undefined. Both genetic and non-genetic risk factors have been reported to dispose an individual to MSTIs [4, 5]. Non-genetic factors, like physical activity and chronic overuse, may be extrinsic contributors to MSTIs. However, the genetic tendency may predispose individuals to a more susceptible condition. In the past years, considerable attention has been focused on genetic basis of MSTIs [6]. Investigators have observed a familial predisposition in MSTIs [7–10]. Besides, genetics were also reported to be associated with athletic performance and rehabilitation [11, 12]. Evidences have been provided to support the association of genetic polymorphisms and susceptibility to MSTIs. Those polymorphisms are mainly located within the collagen-encoding genes, tenascin-C gene, thrombospondin-2 gene, fibrillin-2 gene, matrix proteinase (MMP) gene, and growth differentiation factor 5 gene [13, 14]. Of these genes, COL5A1 is the most extensively studied one.

The COL5A1 gene codes for the α1 chain of type V collagen. Despite type V collagen presents in a smaller amount than other fibrillar collagens, it exerts a crucial role in fibril assembly and inhibition of lateral fibril growth, leading to fewer collagen I fibrils with increased diameters in tendons and ligaments [15]. Literature has reported variants within the 3′-untranslated region (3′-UTR) of COL5A1 gene could modify the secondary structure of the mRNA and mediate its transcript stability [16].

Mokone et al. [17] first reported the rs12722 and rs13946 polymorphisms in CLO5A1 gene and their association with Achilles tendon pathology. Thereafter, multiple replicate studies were conducted with conflicting outcomes. A meta-analysis with nine studies encompassing 1140 cases and 1410 controls indicated that rs12722 polymorphism contributed to tendon-ligament injuries in Caucasians. After that, more than ten studies investigated the association of COL5A1 gene polymorphisms and MSTIs. Enlarging the sample sizes of genetic studies and determining their association with MSTIs will allow investigators estimating which variants predispose to damage of the musculoskeletal system. Therefore, this meta-analysis aimed at collecting and summarizing the existing evidences to elucidate whether COL5A1 gene polymorphisms were associated with MSTIs.

## Methods

### Literature search

An exhaustive literature search of PubMed, Web of Science, EMBASE, Cochrane Library, CNKI, and Wanfang databases was performed to look for studies that reported the association of COL5A1 gene polymorphisms and MSTIs. The terms for literature search included “COL5A1”, “tendon rupture”, “tendon injury”, “ligament injury”, “muscle injury”, “soft tissue injury”, “tennis elbow”, “polymorphism”, “variant”, and “mutant”. The references of eligible articles were also screened for potentially relevant studies. No restriction was set on language or publication date. For non-English and non-Chinese literature, they were translated into English paper by native speaker. The final systematic search was conducted on August, 2021. If necessary, the corresponding author of original articles was contacted for additional information.

### Inclusion and exclusion criteria

The eligible studies should satisfy the following criteria: (1) studies on the association of COL5A1 polymorphisms and MSTIs; (2) cases were confirmed by clinical evaluation and/or other complementary examination; (3) controls were healthy individuals without MSTIs; (4) data were full to evaluate the odds ratios (ORs) and 95% confidence intervals (95%CI).

Correspondingly, the exclusion criteria were as follows: (1) Duplicate data were involved in the studies; (2) conference abstracts, reviews, editorials, or case reports. If multiple studies reported overlapping data, the one with the largest sample size was selected.

### Evaluation of methodological quality

The assessment of study quality was also performed by two authors (RG and ZJ) individually according to the Newcastl-Ottawa Scale (NOS) [18], which included selection (four points), comparability (two points), and exposure (three points). The included studies could be graded as poor, fair or excellent quality based on the following criteria: (1) study quality was poor if one received 0–3 points; (2) study quality was fair if one received 4–6 points; (3) study quality was excellent if one received 7–9 points. Studies with poor quality would be excluded from the final analysis. Any discrepancy was settled by consulting a third reviewer.

### Data extraction

Relevant data were abstracted from qualified studies independently by two investigators (RG and ZJ). The data were first author, publication year, country, ethnicity, gender, study design, diagnosis, genotype distribution of each polymorphism in both groups, result of HWE test [19]. Any divergence was addressed by consulting a third reviewer.

### Statistical analysis

OR and 95%CI were estimated to evaluate the strength of the association. It was assumed that “V” and “v” represented the mutant allele and the wild allele respectively. Therefore, the genotypes could be represented by “VV”, “Vv”, and “vv”. The pooled effect size was calculated respectively for allele contrast of V versus v, homozygote contrast of VV versus vv, heterozygote contrast of Vv vs. vv, dominant contrast of VV + Vv vs. vv, and recessive contrast of VV vs. Vv + vv. The intra-study heterogeneity was evaluated using Q-statistical test and *I*^2^ test. When significant heterogeneity was achieved (*P* < 0.10, *I*^2^ > 50%), the data was merged with the random-effects model. Otherwise, the data were combined with the fixed-effects model. Based on ethnicity (Caucasian, Asian, mixed) and diagnosis (tendon injury, ligament injury, muscle injury), subgroup analyses were performed.

### Sensitivity analysis and publication bias

Sensitivity analysis was performed by sequentially ignoring a single study at a time, which could judge the influence of an individual dataset on the aggregated outcomes. The potential publishing bias was estimated by funnel plots. The data were analyzed by RevMan 5.3 software.

### Functional predictions

Bioinformatics database of HaploReg 4.1 (https://pubs.broadinstitute.org/mammals/haploreg/Haploreg.php) was used to predict the function and interplay of COL5A1 polymorphic sites. String online server was used to examine the network of gene–gene interaction for COL5A1 gene (https://string-db.org/).

## Results

### Literature identification

**Fig. 1:**
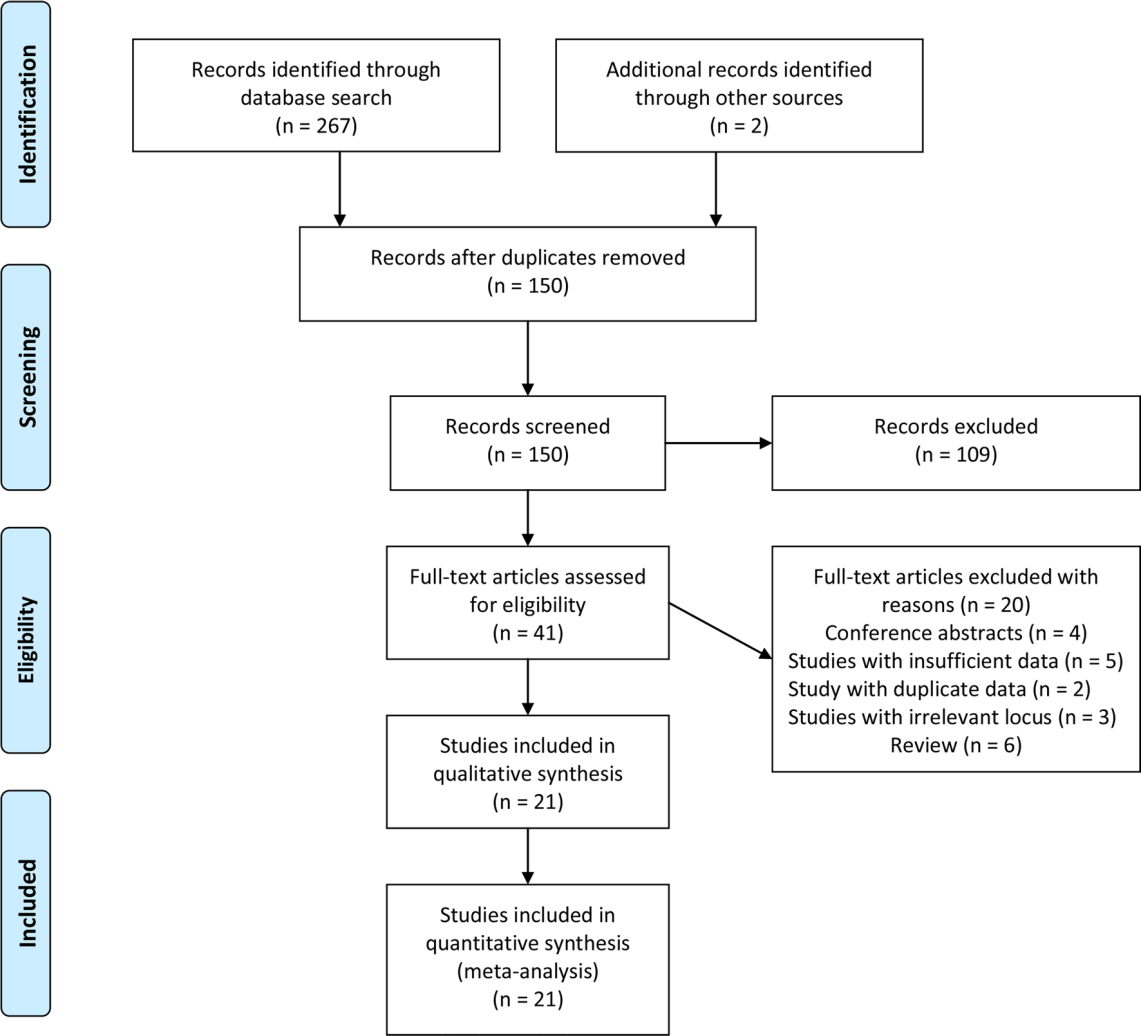
Flow chart of literature identification

The detailed process of literature identification was displayed in Fig. 1. A total of 267 items were obtained from six databases. Two items were yielded via other sources. After the first screen, 119 duplicates were removed. A review of titles and abstracts excluded 109 irrelevant articles. Then, full-text review of 41 articles was completed. Another 20 citations were excluded with reasons. Eventually, 21 articles [17, 20–39] were enrolled in the meta-analysis.

### Main characteristics

The basic characteristics of eligible studies were shown in Table 1. Totally, 21 articles [17, 20–39] were included, of which four articles [27, 28, 31, 36] were cohort studies, one article [22] was cross-sectional study, and the rest 16 articles were case–control studies. Of the included studies, three studies [22, 28, 35] were on Asian population (Chinese, South Korean, Japanese), one study [24] was on a mixed population (Brazilian), and the rest 17 studies were on European population. Twenty studies were published in English with the exception of one in Korean [35]. The publication year ranged from 2009 to 2021. MSTIs in the original studies were divided into three subgroups including tendon injury (rotator cuff tendinopathy, Achilles tendon pathology, Achilles tendinopathy, Achilles tendon rupture, patellar tendinopathy, elbow tendinopathy), ligament injury (anterior cruciate ligament injury, tennis elbow, rotator cuff tear), and muscle injury. Three studies [26, 37, 38] contained two independent cohorts. Of note, a departure from HWE was noted for the rs12722, rs13946, and rs3196378 in some of the studies.

**Table 1:** Main characteristics of included studies

Author	Year	Country	Ethnicity	Gender	Study design	Diagnosis	Case			Control			HWE
							VV	Vv	vv	VV	Vv	vv	
*Rs12722*							TT	TC	CC	TT	TC	CC	
Alakhdar Y	2021	Spain	Caucasian	Both	Case–control	RCTEN	11	31	7	36	47	5	0.04
Alakhdar Y	2020	Spain	Caucasian	Both	Case–control	ETEN	12	20	4	35	58	8	0.02
Altinisik J	2015	Turkey	Caucasian	Both	Cohort	TE	62	46	37	49	85	50	0.31
Brown KL	2017	UK	Caucasian	Both	Case–control	ATP	22	48	29	34	64	27	0.76
Figueiredo EA	2020	Brazil	Mixed	Both	Case–control	RCT	47	94	62	128	248	186	0.01
Haug KBF	2018	Norway	Caucasian	Both	Cohort	PTEN	9	20	4	26	49	18	0.56
Kim H	2015	South Korea	Asian	Both	Case–control	ACLI, RUP	0	15	20	0	8	31	0.48
Leźnicka K	2021	Poland	Caucasian	Both	Case–control	MI	32	12	9	31	16	14	0.01
Longo UG	2018	Italy	Caucasian	Both	Case–control	RCT	33	46	14	74	101	31	0.72
Lulińska-Kuklik E	2018	Poland	Caucasian	Male	Case–control	ACLI	45	66	23	62	107	42	0.74
Miyamoto-Mikami E	2019	Japan	Asian	Both	Cohort	MI	3	52	135	34	400	935	0.26
O'Connell K (I)	2015	South Africa	Caucasian	Both	Case–control	ACLI	72	121	31	65	114	52	0.88
O'Connell K (II)	2015	Poland	Caucasian	Both	Case–control	ACLI	32	44	15	38	75	30	0.54
Raleigh SM	2009	South Africa	Caucasian	Both	Case–control	TEN	29	34	11	25	43	30	0.24
September AV (I)	2009	Australia	Caucasian	Both	Case–control	TEN	17	58	10	74	84	50	0.01
September AV (II)	2009	South Africa	Caucasian	Both	Case–control	TEN	34	47	12	39	55	37	0.07
Sivertsen EA	2019	Norway, Finland	Caucasian	Female	Cohort	ACLI	48	55	15	256	351	124	0.85
Stepien-Slodkowska M	2015	Poland	Caucasian	Both	Case–control	ACLI	48	66	24	53	91	39	0.99
Suijkerbuijk MAM (I)	2019	South Africa	Caucasian	Both	Case–control	ACLI	20	48	25	13	47	36	0.71
Suijkerbuijk MAM (II)	2019	Sweden	Caucasian	Both	Case–control	ACLI	23	36	18	38	47	24	0.20
Zhao D	2020	China	Asian	Both	Cross-sectional	ACLI	8	37	56	6	36	68	0.67
*Rs13946*							TT	TC	CC	TT	TC	CC	
Leźnicka K	2021	Poland	Caucasian	Both	Case–control	MI	26	20	7	28	26	7	0.80
Lulińska-Kuklik E	2018	Poland	Caucasian	Male	Case–control	ACLI	75	49	10	94	102	15	0.07
Mokone GG	2006	South Africa	Caucasian	Both	Case–control	ATP	65	41	5	75	40	14	0.03
September AV (I)	2009	Australia	Caucasian	Both	Case–control	TEN	17	58	9	116	78	50	0.01
Sivertsen EA	2019	Norway, Finland	Caucasian	Female	Cohort	ACLI	70	45	4	410	278	52	0.61
Stepien-Slodkowska M	2015	Poland	Caucasian	Both	Case–control	ACLI	69	57	12	84	88	11	0.06
Zhao D	2020	China	Asian	Both	Cross-sectional	ACLI	39	45	17	32	59	19	0.36
*Rs11103544*							TT	TC	CC	TT	TC	CC	
Figueiredo EA	2020	Brazil	Mixed	Both	Case–control	RCT	147	52	12	182	96	14	0.77
September AV (I)	2009	Australia	Caucasian	Both	Case–control	TEN	55	24	8	140	45	6	0.32
September AV (II)	2009	South Africa	Caucasian	Both	Case–control	TEN	86	35	5	56	29	5	0.63
*Rs71746744*							II	ID	DD	II	ID	DD	
Abrahams Y (I)	2013	South Africa	Caucasian	Both	Case–control	TEN	31	19	1	43	49	9	0.35
Abrahams Y (II)	2013	Australia	Caucasian	Both	Case–control	TEN	25	12	3	45	37	15	0.13
Brown KL	2017	UK	Caucasian	Both	Case–control	ATP	54	45	9	53	67	10	0.08
*Rs3196378*							AA	AC	CC	AA	AC	CC	
Abrahams Y (I)	2013	South Africa	Caucasian	Both	Case–control	TEN	17	42	15	23	50	27	0.99
Abrahams Y (II)	2013	Australia	Caucasian	Both	Case–control	TEN	7	46	10	28	105	42	0.01
Brown KL	2017	UK	Caucasian	Both	Case–control	ATP	34	49	25	33	58	32	0.53
Figueiredo EA	2020	Brazil	Mixed	Both	Case–control	RCT	53	105	53	115	260	189	0.15

V, variant allele; W, wild allele; HWE, Hardy–Weinberg equilibrium; ACLI, anterior cruciate ligament injury; MI, musculoskeletal injury; TE, tennis elbow; RCT, rotator cuff tears; RCTEN, rotator cuff tendinopathy; ETEN, elbow tendinopathy; ATP, Achilles tendon pathology; TEN, Achilles tendinopathy; RUP, Achilles tendon rupture; PTEN, patellar tendinopathy

### Quality assessment

Quality evaluation of the eligible studies was performed by using NOS. Ten studies received 7–9 scores, which were considered to be in excellent quality. The rest eleven studies received 4–6 scores, which were in fair quality (Table 2).

**Table 2:** Quality assessment of included studies

Study ID	Selection				Control for important factor	Exposure			Total
	Adequate definition of cases	Representativeness of cases	Selection of control subjects	Definition of control subjects		Exposure assessment	Same method of ascertainment for all subjects	Non-response rate	
Leźnicka et al. [20]	★	☆	★	★	★☆	★	★	★	7
Alakhdar et al. [21]	★	★	★	★	★★	★	★	★	9
Zhao et al. [22]	★	☆	☆	★	★☆	★	★	★	6
Laguette et al. [23]	★	★	★	★	★☆	★	★	★	8
Figueiredo et al. [24]	★	☆	☆	★	★☆	★	★	★	6
Alakhdar Mohmara et al. [25]	★	★	★	★	★☆	★	★	★	8
Suijkerbuijk et al. [26]	★	★	☆	★	★☆	★	★	★	7
Sivertsen et al. [27]	★	★	★	★	★☆	★	★	★	8
Miyamoto-Mikami et al. [28]	★	★	★	★	★★	★	★	★	9
Haug et al. [31]	★	★	★	★	★☆	★	★	★	8
Lulińska-Kuklik et al. [29]	★	☆	☆	★	★☆	★	★	★	6
Longo et al. [30]	★	☆	☆	★	★☆	★	★	★	6
Brown et al. [32]	★	☆	☆	★	★★	★	★	★	7
Stepien-Slodkowska et al. [33]	★	☆	☆	★	★☆	★	★	★	6
O'Connell et al. [34]	★	☆	★	★	☆☆	★	★	★	6
Kim and Lee [35]	★	☆	☆	★	☆☆	★	★	★	5
Altinisik et al. [36]	★	☆	★	★	★★	★	★	★	8
Abrahams et al. [37]	★	☆	☆	★	★☆	★	★	★	6
September et al. [38]	★	☆	☆	★	★☆	★	★	★	6
Raleigh et al. [39]	★	☆	☆	★	★☆	★	★	★	6
Mokone et al. [17]	★	☆	☆	★	★☆	★	★	★	6

★ The black star represents one socre given

☆ The hollowed star represents one score not given

### Meta-analyses and subgroup analyses

Table 3 summarized the outcomes of overall analyses, and stratified analyses by ethnicity and injury type.

**Table 3:** Associations of *COL5A1* gene polymorphisms and musculoskeletal soft tissue injuries

Polymorphism/genetic models	Effect size of association			No. of cohorts	Test of heterogeneity		Statistical model
	OR	95%CI	*P*		*I*^*2*^ (%)	*P*	
*Rs12722*							
T versus C							
Overall	1.14	1.03–1.28	0.01	21	42	0.02	R
Ligament injury	1.22	1.09–1.38	0.0008	8	0	0.82	F
Tendon injury	1.08	0.89–1.31	0.44	10	61	0.006	R
Muscle injury	1.05	0.66–1.68	0.83	2	57	0.13	R
Caucasian	1.16	1.03–1.31	0.02	17	43	0.03	R
Asian	1.19	0.75–1.89	0.45	3	61	0.07	R
TT versus CC							
Overall	1.33	1.08–1.65	0.008	21	32	0.08	R
Ligament injury	1.52	1.19–1.95	0.0009	8	0	0.79	F
Tendon injury	1.19	0.81–1.77	0.37	10	57	0.01	R
Muscle injury	1.05	0.52–2.13	0.90	2	34	0.22	F
Caucasian	1.38	1.09–1.76	0.008	17	36	0.07	R
Asian	0.98	0.46–2.12	0.97	3	27	0.24	F
TC versus CC							
Overall	1.24	1.03–1.49	0.02	21	37	0.05	R
Ligament injury	1.30	1.05–1.62	0.02	8	0	0.93	F
Tendon injury	1.24	0.85–1.81	0.27	10	61	0.006	R
Muscle injury	0.92	0.66–1.27	0.62	2	0	0.67	F
Caucasian	1.27	1.01–1.59	0.04	17	37	0.06	R
Asian	1.25	0.73–2.14	0.41	3	59	0.09	R
TT + TC versus CC							
Overall	1.28	1.08–1.52	0.005	21	37	0.05	R
Ligament injury	1.37	1.12–1.69	0.002	8	0	0.88	F
Tendon injury	1.24	0.89–1.74	0.21	10	57	0.01	R
Muscle injury	0.93	0.68–1.27	0.65	2	0	0.32	F
Caucasian	1.33	1.08–1.63	0.007	17	33	0.09	R
Asian	1.27	0.73–2.20	0.40	3	63	0.06	R
TT versus TC + CC							
Overall	1.12	0.95–1.35	0.18	21	41	0.03	R
Ligament injury	1.25	1.04–1.51	0.02	8	0	0.86	F
Tendon injury	1.00	0.72–1.37	0.98	10	65	0.002	R
Muscle injury	1.12	0.61–2.05	0.71	2	30	0.23	F
Caucasian	1.14	0.94–1.38	0.20	17	48	0.01	R
Asian	0.97	0.45–2.08	0.93	3	9	0.29	F
*Rs13946*							
T versus C							
Overall	1.09	0.94–1.25	0.25	7	29	0.21	F
Ligament injury	1.19	1.00–1.42	0.05	4	0	0.80	F
Tendon injury	0.90	0.53–1.54	0.71	2	74	0.05	R
Caucasian	1.07	0.92–1.24	0.40	6	37	0.16	F
TT versus CC							
Overall	1.25	0.88–1.76	0.21	7	0	0.53	F
Ligament injury	1.27	0.83–1.96	0.27	4	0	0.47	F
Tendon injury	1.30	0.67–2.54	0.44	2	58	0.12	
Caucasian	1.22	0.83–1.79	0.30	6	1	0.41	F
TC versus CC							
Overall	1.31	0.72–2.42	0.38	7	66	0.007	R
Ligament injury	0.91	0.60–1.39	0.66	4	16	0.31	F
Tendon injury	3.68	1.94–6.98	< 0.01	2	0	0.60	F
Caucasian	1.43	0.70–2.92	0.33	6	69	0.006	R
TT + TC versus CC							
Overall	1.28	0.87–1.89	0.21	7	26	0.23	F
Ligament injury	1.07	0.71–1.61	0.74	4	1	0.39	F
Tendon injury	2.28	1.23–4.23	0.009	2	0	0.78	F
Caucasian	1.41	0.98–2.02	0.06	6	35	0.17	F
TT versus TC + CC							
Overall	1.02	0.69–1.52	0.91	7	76	0.0003	R
Ligament injury	1.32	1.05–1.65	0.02	4	0	0.65	F
Tendon injury	0.54	0.15–1.91	0.34	2	90	0.001	R
Caucasian	0.96	0.62–1.49	0.85	6	79	0.0003	R
*Rs11103544*							
T versus C	0.98	0.61–1.57	0.94	3	75	0.02	R
TT versus CC	0.74	0.31–1.80	0.51	3	53	0.12	R
TC versus CC	0.64	0.35–1.17	0.15	3	0	0.47	F
TT + TC versus CC	0.72	0.41–1.27	0.26	3	40	0.19	F
TT versus TC + CC	1.06	0.65–1.73	0.81	3	66	0.05	R
*Rs71746744*							
I versus D	1.50	1.13–1.99	0.005	3	8	0.34	F
II versus DD	2.04	1.01–4.12	0.05	3	27	0.25	F
ID versus DD	1.21	0.59–2.48	0.61	3	3	0.36	F
II + ID versus DD	1.61	0.81–3.18	0.17	3	27	0.26	F
II versus ID + DD	1.72	1.20–2.46	0.003	3	0	0.67	F
*Rs3196378*							
A versus C	1.21	1.03–1.42	0.02	4	0	0.83	F
AA versus CC	1.46	1.05–2.03	0.03	4	0	0.86	F
AC versus CC	1.25	0.94–1.66	0.13	4	20	0.29	F
AA + AC versus CC	1.45	1.11–1.88	0.006	4	0	0.88	F
AA versus AC + CC	1.16	0.88–1.52	0.28	4	0	0.52	F

OR, odds ratio; CI, confidence interval; F, fixed-effects model; R, random-effects model; ATEN, Achilles tendinopathy; ARUP, Achilles tendon rupture; ACLI, anterior cruciate ligament injury; PTEN, patellar tendinopathy; RCT, rotator cuff tear; TTEN, tibial tendinopathy

#### Association of rs12722 polymorphism and MSTIs

**Fig. 2:**
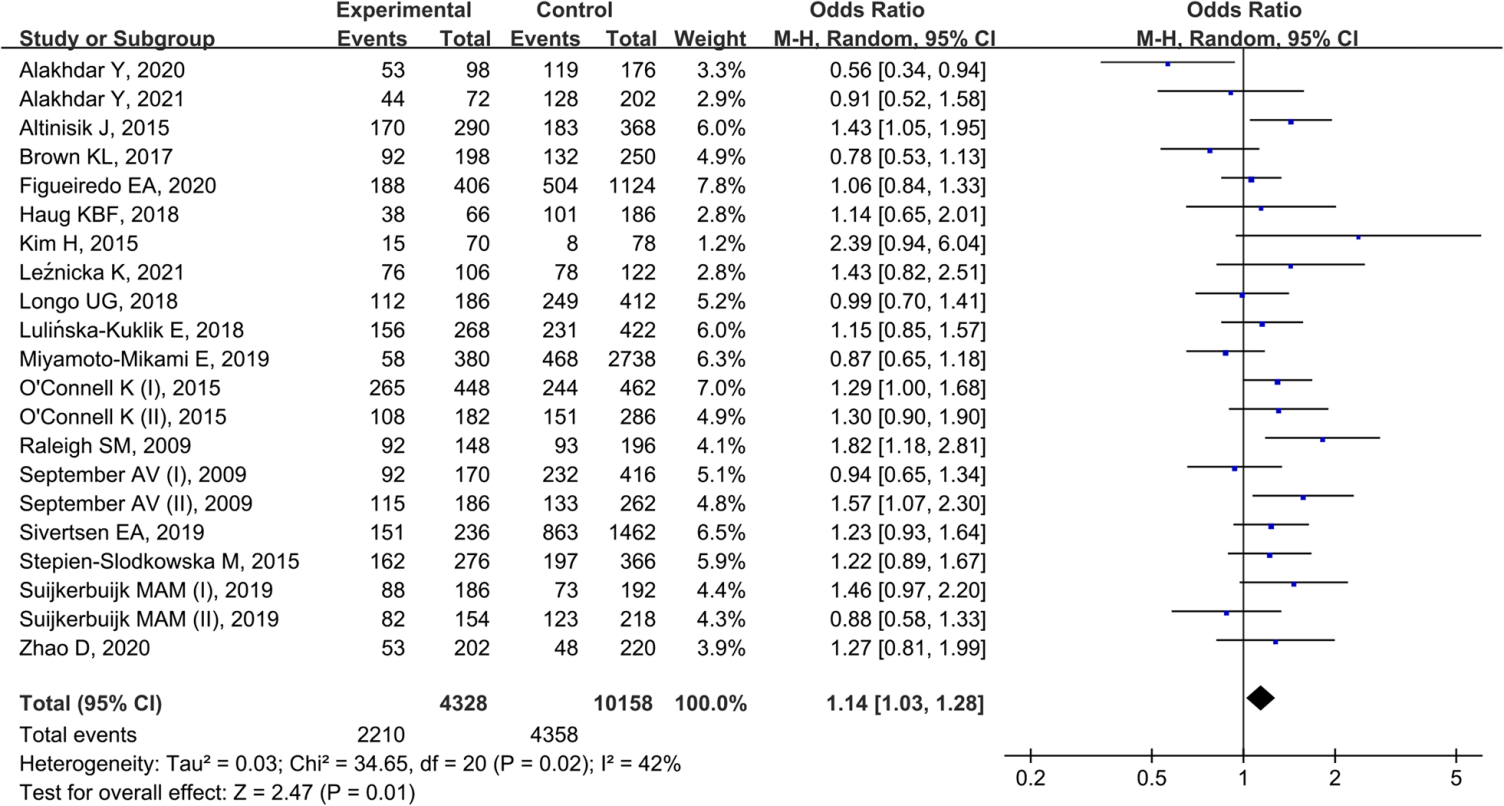
Forest plot of *rs12722* polymorphism and musculoskeletal soft tissue injuries (T vs. C)

Eighteen studies [20–22, 24–36, 38, 39] with 21 cohorts reported the rs12722 polymorphism and vulnerability to MSTIs, encompassing 2164 cases and 5079 controls. Because the heterogeneity was significant, random-effects model was employed. The combined data suggested rs12722 polymorphism was associated with an increased risk to MSTIs under allelic model (T vs. C, OR = 1.14, 95%CI 1.03–1.28, P = 0.01, Fig. 2), homozygote model (TT vs. CC, OR = 1.33, 95%CI 1.08–1.65, *P* = 0.008), heterozygote model (TC vs. CC, OR = 1.24, 95%CI 1.03–1.49, *P* = 0.02), and dominant model (TT + TC vs. CC, OR = 1.28, 95%CI 1.08–1.52, *P* = 0.005).

Stratified analyses by injury type suggested that rs12722 polymorphism was associated with an increased susceptibility to ligament injury under five genetic models. But the association was not found in tendon injury or muscle injury. When stratified by ethnicity, the combined outcomes indicated that s12722 polymorphism was significant associated with MSTIs in Caucasians but not Asians.

#### Association of rs13946 polymorphism and MSTIs

Seven studies [17, 20, 22, 27, 29, 33, 35, 38] reported the rs13946 polymorphism and susceptibility to MSTIs, including 740 cases and 1678 controls. Significant heterogeneity was observed under heterozygote model and recessive model, where the random-effects model was employed. The merged data did not support any association between rs13946 and MSTIs under five genetic models.

Subgroup analyses by injury type suggested that rs13946 was significantly associated with an elevated susceptibility to tendon injury (TC vs. CC, OR = 3.68, 95%CI 1.94–6.98, *P* < 0.01; TT + TC vs. CC, OR = 2.28, 95%CI 1.23–4.23, *P* = 0.009) and ligament injury (T vs. C, OR = 1.19, 95%CI 1.00–1.42, *P* = 0.05; TT vs. TC + CC, OR = 1.32, 95%CI 1.05–1.65, *P* = 0.02). Because only one study was conducted in Asian, stratified analysis by ethnicity was only carried out in Caucasian. The merged data indicated a null association between rs13946 polymorphism and MSTIs in Caucasians.

#### Association of rs11103544 polymorphism and MSTIs

Two studies [24, 38] with 424 cases and 573 controls investigated the association of rs11103544 polymorphism and MSTIs. Substantial heterogeneity was detected under allele model, homozygote model and recessive model, where the random-effects model was used. The pooled data did not support any association between rs11103544 polymorphism and MSTIs.

#### Association of rs71746744 polymorphism and MSTIs

Two studies [32, 37] with 199 cases and 328 controls investigated the association of rs71746744 polymorphism and MSTIs. No heterogeneity was found in five genetic models. The pooled data indicated rs71746744 polymorphism was associated with an increased risk to MSTIs (I vs. D, OR = 1.50, 95%CI 1.13–1.99, *P* = 0.005; II vs. DD, OR = 2.04, 95%CI 1.01–4.12, *P* = 0.05; II vs. ID + DD, OR = 1.72, 95%CI 1.20–2.46, *P* = 0.003).

#### Association of rs3196378 polymorphism and MSTIs

Three studies [24, 32, 37] with 456 cases and 962 controls investigated the correlation of rs3196378 polymorphism and MSTIs. The pooled data indicated a significant association between rs3196378 polymorphism and MSTIs (A vs. C, OR = 1.21, 95%CI 1.03–1.42, *P* = 0.02; AA vs. CC, OR = 1.46, 95%CI 1.05–2.03, *P* = 0.03; AA + AC vs. CC, OR = 1.45, 95%CI 1.111–1.88, *P* = 0.006).

### Sensitivity analysis and publication bias

**Fig. 3:**
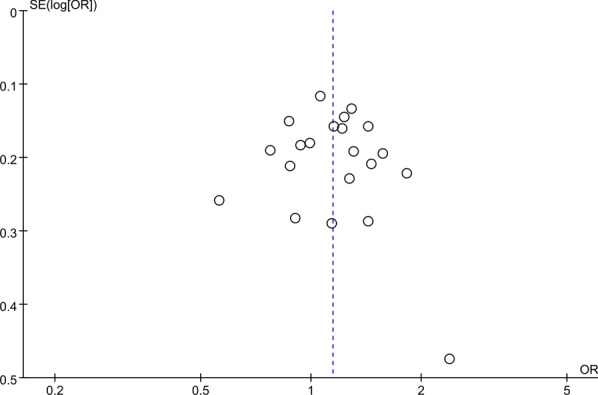
Funnel plot of *rs12722* polymorphism and musculoskeletal soft tissue injuries (T vs. C)

After excluding the studies out of HWE, the OR and 95%CI did not reverse. With sequential removal of each study, the pooled OR and 95%CI of the rest studies did not change significantly, indicating the results were stable. The funnel diagrams did not show obvious sign of dissymmetry, suggesting no significant publication bias (Fig. 3).

### Functional predictions

**Fig. 4:**
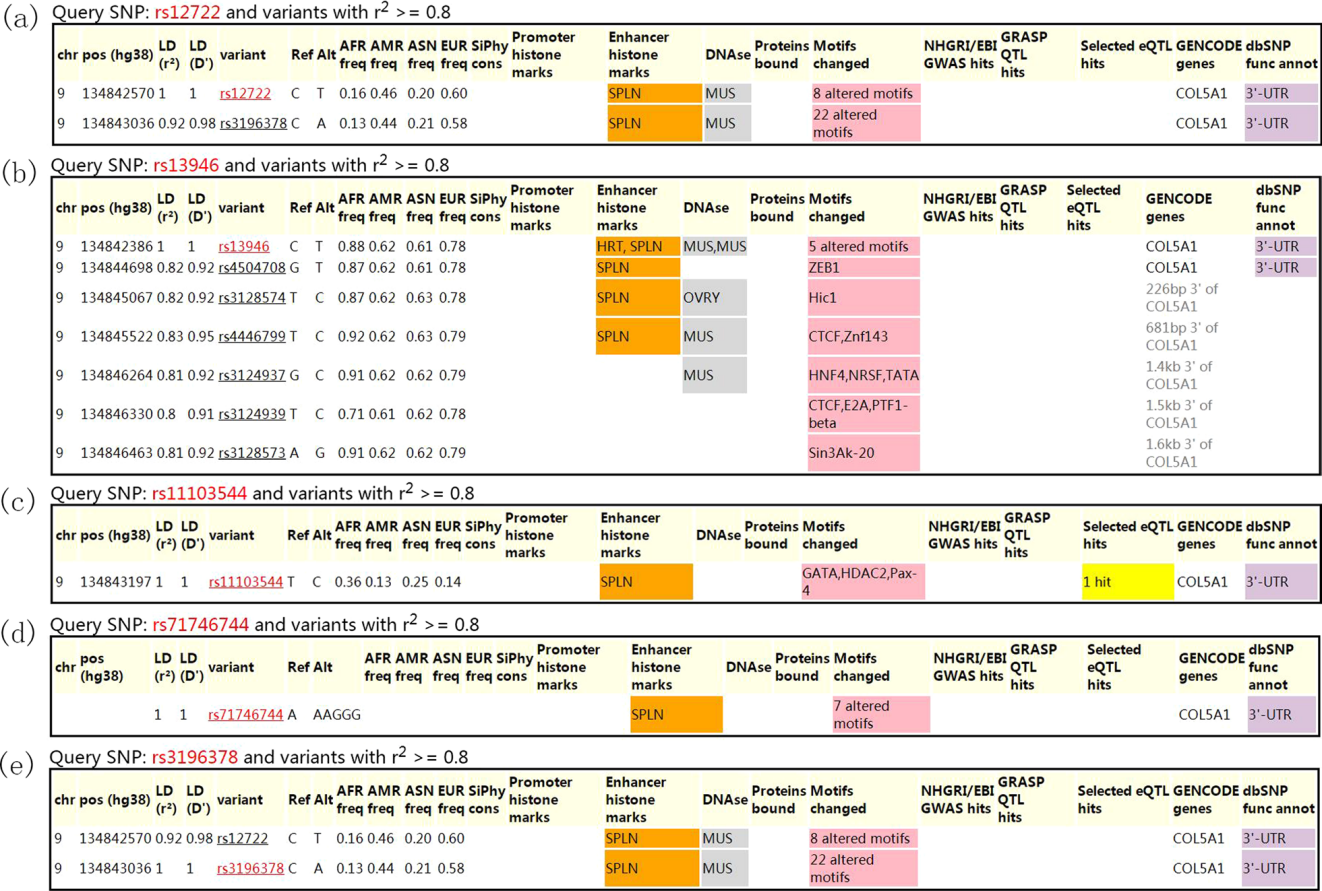
HaploReg view of *COL5A1* gene polymorphisms: **a** *rs12722*; **b** *rs13946*; **c** *rs11103544*; **d** *rs71746744*; **e** *rs3196378*

**Fig. 5:**
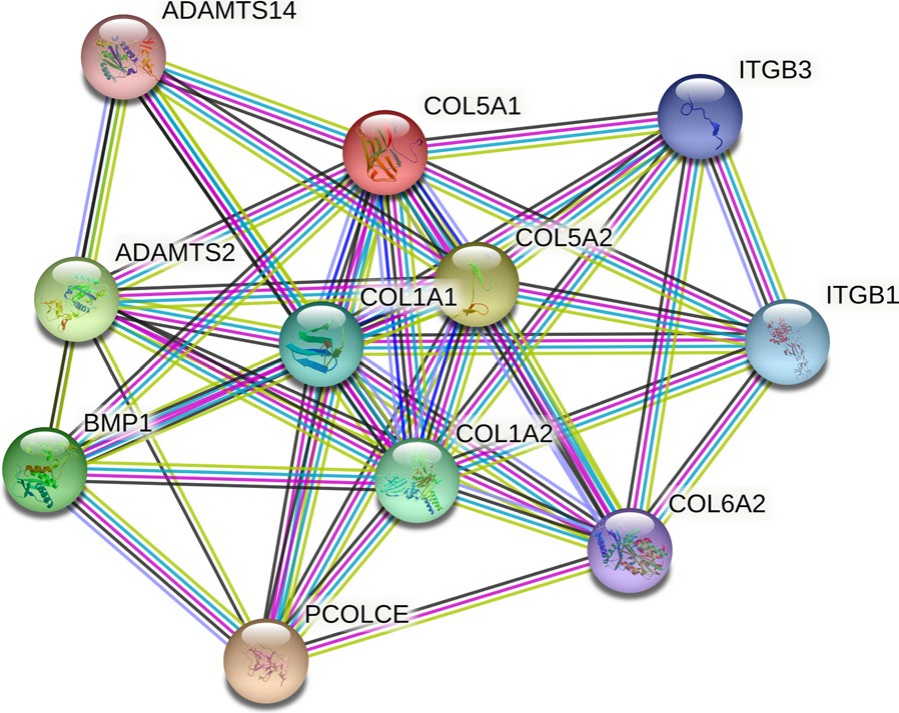
Network of *COL5A1* with its potentially functional partners obtained from String server

The results from HaploReg indicated that rs12722 was in linkage disequilibrium with rs3196378, and rs13946 was in linkage disequilibrium with several other polymorphic sites (Fig. 4). The interactive network of COL5A1 and its partners was presented in Fig. 5. It suggested that COL5A1 might interplay with COL1A1, COL5A2, ADAMTS2, and ADAMTS14.

## Discussion

Knowledge on the pathogenesis of MSTIs may assist the at-risk individuals in reducing the risk of injuries. Genetics are considered to be a non-modified contributor to MSTIs. Evidence from candidate gene studies has added the understanding of the genetic predisposition to MSTIs. Nogara et al. reported that rs4986938 polymorphism in ER-β gene contributed to posterior tibial tendinopathy in the Brazilian population [40]. Diniz-Fernandes et al. found that MMP-1 and MMP-8 gene polymorphisms promoted increase and remodeling of the collagen III and V in posterior tibial tendinopathy [41]. Artells et al. reported that rs2289360 variant in elastin gene is a potential biomarker for ligament injuries in elite soccer [42]. Insulin-like growth factor 2 and elastin gene polymorphisms were reported to be associated with the degree and recovery time for tendon injuries [43]. Besides, predictive DNA profiling might help athletes to maximize utilization of their potential and improve performance in sports [44]. COL5A1 gene is of particular interest among the candidate susceptible genes for MSTIs. However, the role of COL5A1 gene polymorphisms in MSTIs susceptibility remained the subject of debate.

Reasons like diverse recruitment criteria, varied characteristic of participants, different sample size, heterogeneous ancestries and genders, may result in the inconsistency. Given meta-analysis is a powerful approach to combine data from independent studies and explain heterogeneity, this study was conducted to make a more precise estimation of the correlation of COL5A1 gene polymorphisms and MSTIs. The overall analyses supported that rs12722, rs71746744, and rs3196378 polymorphisms were associated with an increased risk to MSTIs. But the association was not identified in rs13946 or rs11103544 polymorphism. Of note, the positive association appeared to be significant in Caucasians but not Asians for rs12722 polymorphism. A detailed analyses by injury type showed that rs12722 polymorphism was associated with ligament injury, but not tendon injury or muscle injury. For rs13946 polymorphism, it appeared to be associated with tendon injury and ligament injury. It is worthwhile mentioning that the variant T of rs12722 is more frequent in Europeans (MAF: 0.60) than in Asians (MAF: 0.20, Fig. 6). Therefore, the inconsistent outcomes between Asians and Caucasians may be attributable to differences in genetic background.

Based upon the current findings, future works should be focus on rs12722 and rs13946 polymorphisms. As each individual has a unique genetic profile, genetic screening tools might be designed to identify individuals predisposed to MSTIs, thus enabling implementation of preventive strategies for them. Correspondingly, taking preventive measures might reduce the incidence of MSTIs and its cost [45]. While, it should be pointed out that none of the genetic polymorphisms could solely decide the injury risk. Therefore, multifactorial models should be developed to predict the risk of MSTIs [46].

Collagen is best known as the principal tensile element of connective tissues like tendons, ligaments, and cartilage [47]. Type V collagen is composed of several isoforms, but the key isoform is consisted of two α1 chains and one α2 chain, which are encoded by COL5A1 and COL5A2 genes, respectively [36]. Literature has reported that mutation of COL5A1 gene is associated with Ehlers-Danlos syndrome (EDS), a genetic disorder mainly characterized with irregular collagen fibrils. Individuals with EDS exhibit hyperelasticity and laxity in a variety of tendon-ligament tissues, indicating that COL5A1 gene is responsible for the adequate function of soft connective tissues [48]. Wenstrup et al. [49] reported that heterozygous mice with COL5A1 gene showed tremendously defective collagen fibril formation and increased fibril diameter, leads to the connective tissue dysfunction. Goncalves-Neto et al. [50] observed an increased type V collagen and a reduced type I collagen in injured tendons. Based upon the abovementioned evidences, it is reasonable that variants in COL5A1 gene may contribute to MSTIs.

The five studied loci were located in the 3’-UTR of COL5A1 gene. Despite 3’-UTR has a noncoding character, mutations within this region may modify the secondary structure of mRNA and protein features [51]. Indeed, Laguette et al. [16] had reported that the luciferase activity of the C-allele significantly decreased than that of the T-allele for rs12772 polymorphism, and there was an increase in COL5A1 mRNA stability in the individuals with tendinopathic disorder. Collins et al. [15] reported that rs12772 variant might cause an altered amount of type V collagen production, which altered the fibril architecture and mechanical properties. Rs3196378 and rs11103544 were located in the downstream of rs12722, and they spanned miRNA binding sites. Therefore, the two variants potentially had a functional significance in MSTIs [38].

To investigate the interaction effects between polymorphic sites, functional predictive analysis was performed. The results from HaploReg indicated that rs12722 was in linkage disequilibrium with rs3196378, and rs13946 was in linkage disequilibrium with several other polymorphic sites (Fig. 4). In addition, interactions of COL5A1 with other gene might play a role in the present genetic polymorphisms. Functional prediction also suggested that COL5A1 might be involved in the gene–gene interaction with COL1A1, COL5A2, ADAMTS2, and ADAMTS14, which have been reported to be associated with MSTIs [20, 52, 53]. Further studies are encouraged to confirm these interactions in more details.

Of note, Lv et al. [54] had published a similar meta-analysis on this topic. Compared with the previous one, the current meta-analysis had notable improvements. First, the previous study employed a model-free approach to analyze the association of rs12722 polymorphism and MSTIs. Concerning the inheritance models were complex in MSTIs, this study examined five genetic models to explore the underlying association. Second, some most recently published evidences were added into this study, which greatly enlarged the literature number and sample size. Therefore, the statistical power of the pooled results became much stronger. Third, subgroup analysis by ethnicity and injury type was conducted, and an ethnicity-specific effect was found on rs12722 polymorphism and MSTIs. Fourth, for rs13946, rs11103544, rs71746744 and rs3196378 polymorphisms, no combined study had examined their association with MSTIs.

However, several potential drawbacks could not be overcome in this study. First, although subgroup analysis was performed, the heterogeneity in some contrasts still could not be well addressed.

The heterogeneity might be explained by diversity of injury types, differences in sequencing methods, variance of ethnic origins, and differences in the selection of participants. Heterogeneity should be considered when interpreting the findings, and future studies should be focused on more homogenous groups of patients. Second, the number of studies of rs13946, rs11103544, rs71746744 and rs3196378 polymorphisms was small. The statistical power might not be strong enough to explore the relationship of the four polymorphisms and MSTIs. Third, clinical heterogeneity, such as age, sex, lifestyle, mechanism of injury, physical or occupational activity, and other potential confounding factors, could not be managed, which might distort the outcomes. Fourth, because the ethnicity subgroup analyses were restricted to European, Asian and Brazilian populations, the results are only applicable to such ancestry groups. Fifth, several of the included studies were out of HWE, which could be caused by population stratification, genotyping errors, and selection bias in the recruitment of controls [55]. Last, because of the included studies were observational studies, the evidence level presented in this meta-analysis was relatively low.

## Conclusions

Taken together, the current meta-analysis supports that rs12722 is associated with an elevated susceptibility to ligament injury, especially in the Caucasian population. Rs13946 polymorphism appears to increase the risk to tendon and ligament injuries. Rs71746744 and rs3196378 polymorphisms have a tendency to confer an elevated risk to MSTIs. However, no relevance is found between rs11103544 polymorphism and MSTIs. Given limitations in this meta-analysis, it is encouraged to verify these findings with complementary larger and well-designed prospective studies.

## Data Availability

The datasets are available from the corresponding author on reasonable request.

## Abbreviations

NOS: Newcastle–Ottawa Scale
OR: Odds ratio
95%CI: 95% Confidence interval
COL5A1: Collagen type V α1
MSTIs: Musculoskeletal soft tissue injuries
